# Correlation between Macrophage Polarization and PD-L1-Related Tumor Microenvironmental Alteration and Metastasis in Pancreatic Ductal Adenocarcinoma

**DOI:** 10.1155/2023/7971306

**Published:** 2023-03-06

**Authors:** Jiangning Gu, Feng Gao, Bin Fan, Shiqi Xu, Haifeng Luo, Jie Zhang, Qi Jiang, Chenqi Wang, Guang Tan, Wenjing Qi, Jian Du, Siqian Huang, Xiaohong Sun, Dan Chen

**Affiliations:** ^1^Department of Hepatobiliary Surgery, the First Affiliated Hospital of Dalian Medical University, Dalian, Liaoning, China; ^2^Department of Digestive Endoscopy, General Hospital of Northern Theater Command, Shenyang, Liaoning, China; ^3^Department of General Surgery, the First Hospital of Northwest University (Xi'an No. 1 Hospital), Xi'an, China; ^4^Department of Oncology, the First Affiliated Hospital of Dalian Medical University, Dalian, Liaoning, China; ^5^Department of Pathology, the First Affiliated Hospital of Dalian Medical University, Dalian, Liaoning, China; ^6^Department of Nursing, the First Affiliated Hospital of Dalian Medical University, Dalian Medical University, Dalian, Liaoning, China

## Abstract

Pancreatic ductal adenocarcinoma (PDAC) is a highly aggressive malignancy with a poor prognosis; nearly 80% patients have regional or distant metastasis when diagnosed. Tumor microenvironment (TME) alteration and epithelial-to-mesenchymal transition (EMT) have been reported to play a key role in cancer metastasis. However, the correlation between TME and EMT was poorly studied in PDAC. This study aims to explore the correlation between EMT markers and TME alteration, mainly including macrophage polarization and PD-L1 expression change, in primary and metastatic PDAC tissues by immunohistochemistry. The results indicated that macrophage polarization was found in metastases with the number of M1 macrophages (CD86^+^) decreased and M2 (CD163^+^) increased, though PD-L1 expression did not have a significant alteration. Compared to primary tumors, E-cadherin was significantly lower, while snail was higher, while no difference was found with N-cadherin and ZEB1. Correlation analysis indicated that snail, but not ZEB1, E-cadherin, or N-cadherin, was highly correlated with macrophage polarization. To conclude, the number of CD86^+^ M1 macrophages was increased while CD163^+^ M2 macrophages decreased in metastases, with no significant alteration of PD-L1 expression compared to primary tumors. EMT markers—Snail and E-cadherin—but not ZEB1 or N-cadherin, were found to be higher/lower in metastases, which mean that EMT played an important role in PDAC metastasis. Further analysis indicated that snail was highly correlated with M1 to M2 macrophage polarization, which prompted that EMT may be one reason for macrophage polarization induced TME alteration in PDAC metastasis.

## 1. Introduction

Pancreatic ductal adenocarcinoma (PDAC) remains an exceedingly aggressive malignancy with increased incidence, and the 5-year survival rate is only around 11% according to the latest statistics [1]. Early metastasis is one of the main reasons for the poor prognosis. Comprehensive therapy performs a necessary position in the treatment of PDAC besides surgical resection. However, there are no extensively accepted targeted drugs, and common chemotherapeutics are still the mainstream treatment with unsatisfied results.

Metastasis is the signal of poor prognosis of malignant tumors including PDAC. The advent of metastasis means cancer cells have to breach special immune barriers. Tumor microenvironment alteration provides an appropriate condition for cancer metastasis after a complex of regulation. Tumor microenvironment is a complex system composed of cells that evolve with tumor cells and provide support in the process of malignant transformation. Macrophage is an important group in the tumor microenvironment, which can prevent and promote tumor growth. Tumor-associated macrophages (TAMs) have been classified into tumor-inhibiting (M1) and tumor-promoting (M2) macrophages. The M1 subtype could be polarized into M2 under different conditions. Studies have indicated that macrophage polarization from M1 to M2 is correlated with poor prognosis in various types of cancer [2, 3]. However, the relationship between metastasis and macrophage polarization has not been fully elucidated.

Blockade of programmed death-1 (PD-1) or programmed death ligand-1 (PD-L1) has become a new immunotherapy and achieved exciting results in some types of cancer, such as melanoma [4], nonsmall cell lung cancer [5], and hepatocellular carcinoma [6]. However, there were only sporadic case reports and clinical trials, albeit with varying effects in PDAC. Some studies have already pointed out that PD-L1 and PD-1 were differentially expressed in primary and metastatic sites [7, 8]. Unfortunately, this hypothesis has not yet been validated in PDAC, and the potential mechanisms need to be further elucidated. Thus, the effect of immune-checkpoint inhibitors (ICIs) may depend on different tumor microenvironment statuses, and targeting metastatic patients through PD-1/PD-L1 inhibition may be an effective therapeutic strategy under this circumstance.

Epithelial-to-mesenchymal (EMT) is a cellular remodeling process associated with a sequence of biological processes, including tumor growth, invasion, metastasis, and chemotherapy. It has additionally been reported that EMT involves immune suppression, evasion, and tolerance [9]. We hypothesized that the EMT and tumor microenvironment status of the primary tumor and metastatic site of PDAC were different. The result might provide a theoretical foundation for the treatment of metastatic PDAC.

## 2. Materials and Methods

### 2.1. Patients and Specimens

A total of 50 cases of PDAC diagnosed by pathology from January 2013 to December 2020 in the Pathology Department of the First Affiliated Hospital of Dalian Medical University were selected. All patients did not receive any other anticancer treatment, such as chemotherapy or radiotherapy, before surgery. Among the 50 cases, 33 were primary tumors with paired regional metastatic lymph nodes, 9 cases were collected during surgical resection with primary tumors and oligometastases or micrometastases, which were failed to evaluate before surgery but have the probability to get radical resection after exploratory laparotomy and evaluated intraoperatively. The remaining 8 only have hepatic metastasis through biopsy in order to reach correct diagnosis for future treatment direction. A total of 92 lesions were selected from 42 primary tumor specimens and 50 metastatic tumor specimens. The clinicopathological parameters of patients with PDAC were collected, including gender, tumor size, degree of tumor differentiation, neural invasion, vascular invasion, TNM stage, serum CA19-9 level, and serum CA125 level. The degree of tumor differentiation was graded according to the grading standard of PDAC in the 5^th^ edition of the “WHO Classification of Tumors of the Digestive System” [10]. Patients with PDAC were clinically staged according to the 8th edition of the American Joint Cancer Society (AJCC) of pancreatic cancer staging [11]. All cases were confirmed and reviewed by two senior pathologists. This research was approved by the Medical Ethics Committee of the First Affiliated Hospital of Dalian Medical University. All specimens were collected with consent from patients or their relatives.

### 2.2. Immunohistochemistry

Envision two-step approach was taken in immunohistochemistry. The following antibodies were used: rabbit monoclonal anti-CD86 antibody (Cell Signaling Technology Cat# 91882), rabbit monoclonal anti-CD163 antibody (Gene Tech Cat# GT2077), rabbit monoclonal anti-PD-L1 antibody (Cell Signaling Technology Cat# 13684), rabbit monoclonal anti-E-cadherin antibody (Gene Tech Cat# GT210702), rabbit monoclonal anti-N-cadherin antibody (Cell Signaling Technology Cat# 13116), rabbit monoclonal anti-ZEB1 antibody (Cell Signaling Technology Cat# 70512), and rabbit polyclonal anti-Snail antibody (HuaBio, Cat# ER1706-22).

First, formalin-fixed and paraffin-embedded tissues were cut into 4-µm sections. After being deparaffinized and rehydrated, the sections were heated with EDTA antigen retrieval solution in a pressure pot for 3 min and cooled at room temperature. Then, 3% H_2_O_2_ solution was used to block endogenous peroxidase activity for 10 minutes. After washing with phosphate buffered solution (PBS), the tissue sections were incubated with specific antibodies at an appropriate dilution ratio for 1 h at room temperature. After washing with PBS again, the tissue sections were then incubated with horseradish peroxidase-conjugated secondary antibody (PV6000D, Zhongshan Golden Bridge Biotech Co. Ltd) at room temperature for 20 min. The isotype was used as the control. The antigen-antibody complexes were visualized using DAB and counterstained with hematoxylin.

### 2.3. Evaluation of Immunochemistry

All slides were reviewed and evaluated by senior pathologists with no clinicopathological information in a double-blinded manner. The expression of CD86 was detected on the cell membrane of M1 macrophages. CD163 was located on the cell membrane or in the cytoplasm of M2 macrophages. In this study, we used CD86 and CD163 as markers to assess M1 and M2 macrophages distribution in primary tumors and metastases. CD86^+^ and CD163^+^ macrophages were analyzed as follows. Five most representative spots were chosen from ×100 fields per slide. The number of positive cells was counted under high magnification (×400) in the tumor nest and stroma area. The mean number of M1/M2 macrophages was calculated and recorded. The medians were calculated, respectively, and those higher than the median were designated as the high expression group, and those lower than the median were designated as the low expression group.

Positive PD-L1 expression was designated when the membrane of tumor cells was stained in comparison with that of the negative control. The tumor proportion score (TPS) was defined as the number of PD-L1-staining tumor cells divided by the total number of viable tumor cells multiplied by 100. Then, the positivity of PD-L1 was defined as scores above 0.

The final scores for E-cadherin, N-cadherin, Snail, and ZEB1 were calculated as follows: ×400 magnification fields were chosen randomly, and the staining intensity was marked as 0, 1, 2, and 3 for negative, weak, intermediate, and strong staining, respectively. Then, in each field, the percentage of positive cells was scored as follows: 0 (<5%), 1 (5%–25%), 2 (26%–50%), 3 (51%–75%), and 4 (>75%). The IHC score, calculated by multiplying the two scores, ranged from 0 to 12.

### 2.4. Statistical Analysis

Statistical analysis was performed by SPSS (Version 26, Chicago, USA). Mann–Whitney *U* test was used to compare the expression of CD86, CD163, PD-L1, E-cadherin, N-cadherin, Snail, and ZEB1 between the primary and metastases of PDAC since they did not fit the normal distribution. Spearman's rank correlation analysis was used to analyze the correlation between EMT (E-cadherin and Snail) and macrophage markers (CD86 and CD163) in metastases of PDAC. The chi-square test and Fisher's exact test were used to analyze the correlation between the targeted protein expression and clinicopathological parameters in patients with PDAC. *P* < 0.05 was set as a significant difference for all statistical analyses.

## 3. Results

### 3.1. Demographic Characteristics

The clinicopathological characteristics of the patients have been summarized in Table 1. Among the 42 primary tumors of PDAC, 22 cases were male and 20 were female. The average tumor diameter was 4.1 cm. 20 cases were high to moderate differentiation and 22 were low differentiation. 26 cases were validated with vascular invasion (61.9%) and 41 cases with perineural invasion (97.6%). According to the 8^th^ AJCC TNM stage, the clinical stage was as follows: stage I (0 cases), stage II (26 cases), stage III (7 cases), and stage IV (9 cases). All the cases were defined after surgical resection. Serum CA199 level was elevated in 38 cases (90.5%), serum CA125 level was elevated in 14 cases (33.3%). 8 cases only have metastases from preoperative biopsies and were confirmed as PDAC according to immunohistochemistry and multidisciplinary diagnosis.

### 3.2. Correlation between Macrophage Polarization and Metastasis of Pancreatic Ductal Adenocarcinoma

**Figure 1:**
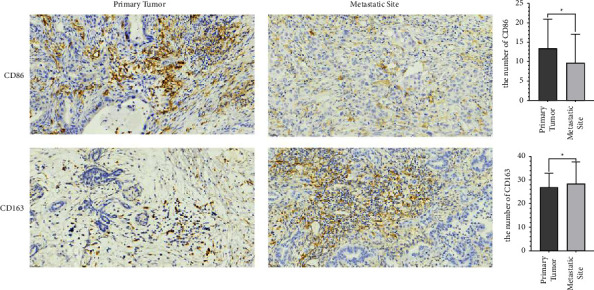
Representative immunohistochemical image and the number of CD86 and CD163 in primary tumors and metastatic site.

The mean number of infiltrated CD86^+^ M1 macrophages was 11.6 (2–31.2) in primary tumors and 8.4 (1.4–27.8) in metastases (*P* = 0.012) Figure 1, Table 2. While the mean number of infiltrated CD163^+^ M2 macrophage was 27.1 (7.6–38.2) in primary tumors and 31 (8.4–45) in metastases (*P* = 0.03) (Figure 1, Table 3. These results demonstrated that the number of M1 macrophages decreased while M2 macrophages increased, which could be regarded as evidence for macrophage polarization during the process of PDAC metastasis. No correlation was observed between the number of CD86/CD163 and clinical parameters in our cohort (Table 4).

### 3.3. Correlation between PD-L1 Expression and Metastasis of Pancreatic Ductal Adenocarcinoma

**Figure 2:**
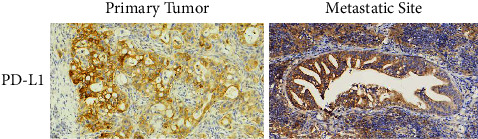
Representative immunohistochemical image of PD-L1 in primary tumors and metastatic site.

According to the tumor proportion score (TPS), the expression of PD-L1 in primary tumors ranged from 0 to 50%, while from 0 to 20% in metastases (Figure 2). There was no significant difference between these two groups (*P* = 0.468) (Table 5). Correlation analysis indicated that PD-L1 level was highly correlated with TNM stage (*P* = 0.031) (Table 6), which demonstrated that the early stage of PDAC had a relatively higher-level PD-L1 score.

### 3.4. Correlation between EMT and Metastasis of Pancreatic Ductal Adenocarcinoma

**Figure 3:**
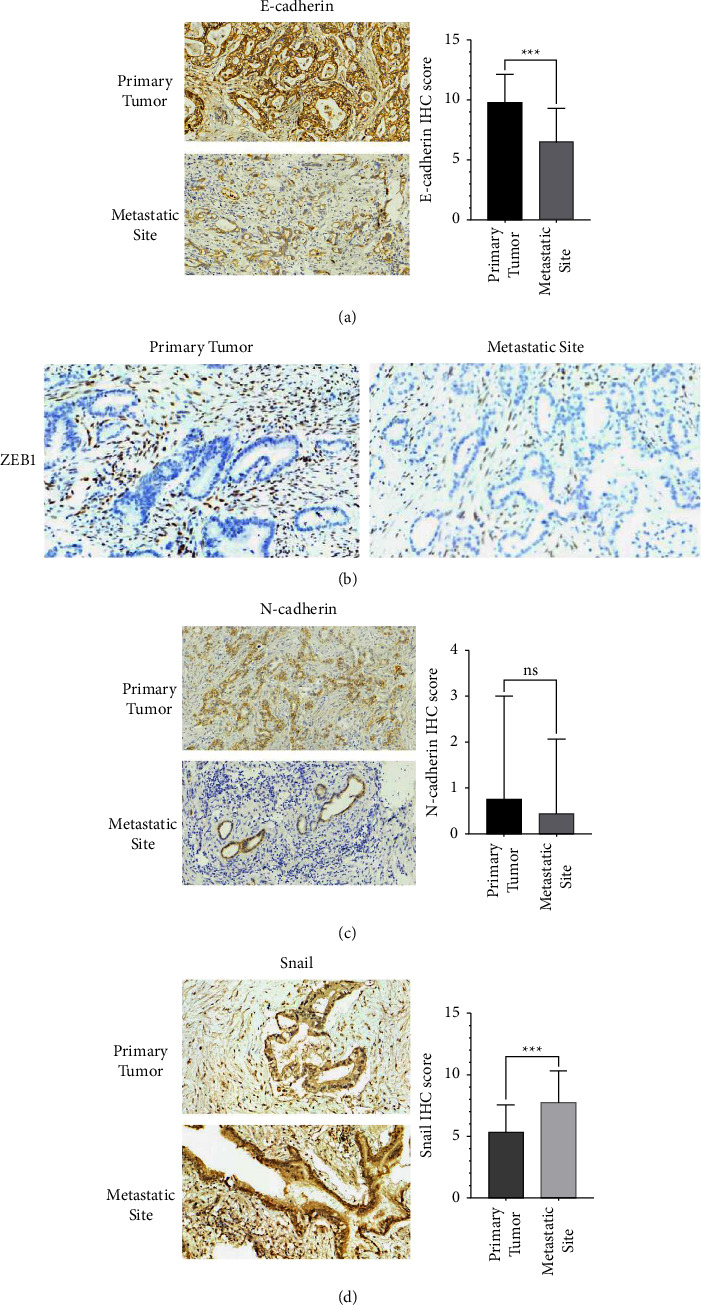
Immunohistochemistry and quantification of EMT markers in in primary tumors and metastatic site. (a) Representative immunohistochemical image of E-cadherin in primary tumors and metastatic site. (b) Representative immunohistochemical image of ZEB1 in primary tumors and metastatic site. (c) Representative immunohistochemical image of N-cadherin in primary tumors and metastatic site. (d) Representative immunohistochemical image of Snail in primary tumors and metastatic site. ^∗∗∗^*P* < 0.001; ns, not significant.

E-cadherin, the most commonly used epithelial marker, was significantly decreased in metastases (*P* < 0.001) (Figure 3(a)). Three mesenchymal markers- N-cadherin, Snail, and ZEB1 were also detected in primary tumors and metastases. Results indicated that ZEB1 was nearly negative in epithelial cells but strongly positive in interstitial cells, which were not used for further analysis (Figure 3(b)). There was no significant difference of N-cadherinexpression between these two groups (Figure 3(c), *P* = 0.698). However, Snail expression was significantly higher in metastases compared to primary tumors (Figure 3(d), *P* < 0.001). The above results indicated that epithelial marker E-cadherin was decreased in metastases, while mesenchymal marker Snail was upregulated, which further validated that EMT was highly associated with cancer metastasis in PDAC.

Correlation analysis indicated that N-cadherin was highly related with tumor differentiation and CA125 level, while Snail was related to clinical TNM stage. This was also in accordance with the current results, which show that higher mesenchymal markers are associated with aggressive phenotypes (Table 7).

### 3.5. Correlation between EMT and Macrophage Polarization

**Figure 4:**
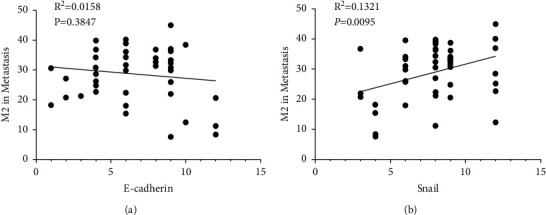
Spearman correlation analysis between the number of M2 macrophages infiltration in metastatic site and EMT markers. (a) There was no significant correlation between the number of M2 macrophages infiltration and E-cadherin in metastases. (b) Significant correlation was found between the number of M2 macrophages infiltration and E-cadherin in metastatic site.

In order to clarify whether EMT was correlated with macrophage polarization, we analyzed the relationship between EMT markers (E-cadherin and Snail) and the number of M2 macrophages infiltration in metastases. The results indicated that, though no clear relationship was found between E-cadherin and CD163^+^ cells alteration, Snail was significantly correlated with M2 macrophage infiltration in metastases (Figure 4), which indicated that Snail may play a role in macrophage polarization in PDAC metastasis.

## 4. Discussion

One of the reasons for the poor prognosis of PDAC is the early metastasis to regional or distal organs [12]. Thus, investigation of the potential mechanisms for PDAC metastasis should assist help to come throughout the barrier of the cancer therapy. As time progresses and we learn more, researchers now not solely pay interest to the cancer cells themselves, however, additionally the interstitial cells around the cancer cells, such as fibroblasts and immune cells. These cells consisted a new environment supporting tumor development, which is known as the tumor microenvironment (TME). In TME, the crosstalk between most cancer cells and interstitial cells could arise many biological processes like tumor growth, metastasis, and therapeutic resistance [13].

According to the latest studies, the immune cells are double-edged swords with the feature of each tumor promoting and suppression [14]. The immune cells originally could play an important role in the procession of recognition, initiation of inflammation, and antitumor responses in tumorigenesis. Among the immune cells, macrophages play a necessary position in the process of antitumor responses. However, there are two subtypes of macrophages. M1 macrophages have antitumor effects, while M2 macrophages could promote tumor progression by different regulatory mechanisms, such as inflammation promotion and immune adaptation [15–17]. The transition from M1 to M2 macrophages used to be named as macrophage polarization which has been validated to be associated with tumor invasion and metastasis [18]. The hypothesis indicated that during the process of metastasis, the TME will change to create an appropriate circumstance for cancer cells colonization. Among these, macrophage polarization is an important event. Our study was consisted with this, compared to primary tumors, the number of M1 macrophages decreased while M2 macrophages increased through immunohistochemistry validation in clinical samples, which imply macrophage polarization was once correlated with PDAC metastasis.

Except tumor-associated macrophages, PD-L1 and PD-1 are additionally hot spots in cancer research. The ligand-receptor combination could restrict the function of CD8^+^ T cells. Targeting PD-L1 has become an effective treatment in some solid malignancies [7, 19], however, not in PDAC. One of the reasons is that PD-L1 expression is vulnerable in PDAC tissues, however, few studies have investigated its expression in metastases. Schneider et al. validated that PD-L1 expression was associated with the presence of lymph node metastasis in head and neck squamous cell carcinoma, while Wei et al. stated that the expression of PD-L1 in liver metastases was higher than in primary tumors of colorectal cancer [19]. We considered that if PD-L1 was higher in metastases, this might also provide a new theoretical approach for anti-PD-L1 therapy in advanced PDAC. In our study there was no significant difference of PD-L1 expression between primary tumors and metastases; however, we discovered PD-L1 is relatively higher in early PDAC patients (stage I and II); this reminded us that whether or not anti-PD-L1 may hardly have impact in late stage of PDAC due to immune suppression, which needs further investigation.

As we discussed above, TME alteration may create an appropriate environment for cancer cells metastasis. Except for TME alteration, cancer cells also adapted themselves to the new microenvironment. EMT, a vital form of cell remodeling, has been reported with cancer metastasis [20], however, whether EMT was once correlated with TME, especially macrophage polarization and PD-L1 expression, was poorly investigated in PDAC. In our study, we unexpectedly found this transformation in metastases, with E-cadherin decreased and Snail increased. We further studied the relationship between TME and EMTand found that, though there was no correlation between EMT markers and PD-L1 expression, the mesenchymal marker Snail was highly related to M2 macrophage infiltration in PDAC metastasis. These results indicated that cell remodeling and microenvironment alteration are dynamic processes.

Our study has some limitations. First, no matter if it is EMT or tumor-associated macrophages, there are many markers; it is hard to finish all of them, and the present biomarkers could not represent the whole group, while not only immune cells act as a role in tumorigenesis, TME is a complicated content. Second, the small size of the matched distant metastases cohorts may be another defect due to the difficulty of sample collection. Third, our study was conducted only in clinical samples, without in vivo or in vitro validation.

In summary, macrophage polarization was found in metastases, with the number of CD86^+^ M1 macrophages reduced and CD163^+^ M2 macrophage increased. E-cadherin was significantly lower in metastases, while mesenchymal marker Snail was higher. Correlation analysis indicated that Snail was highly related to macrophage polarization, which reminded us that TME was may be associated with EMT in PDAC metastasis.

## 5. Conclusion

Compared to primary tumors, the number of CD86^+^ M1 macrophages was decreased, while CD163^+^ M2 macrophages increased in metastases with no significant alteration of PD-L1 expression. EMT markers, Snail and E-cadherin were found to be higher/lower in metastases, whichmeans that EMT played an important role in PDAC metastasis. Further analysis indicated that Snail was highly correlated with M2 macrophage infiltration, which prompted that EMT may be one reason for macrophage polarization associated TME alteration in PDAC metastasis.

## Figures and Tables

**Table 1:** Basic clinicopathologic data for primary pancreas tumor.

Characteristics	Primary tumor
Gender (no. (%))	
Male	22 (52.4)
Female	20 (47.6)

Maximum diameter of tumor	
Mean (cm (SD))	4.1 (1.9)
Range (cm)	1.8–10

Grade (no. (%))	
High/moderate differentiation	20 (47.6)
Poor differentiation	22 (52.4)

Vascular invasion (no. (%))	
Yes	26 (61.9)
No	16 (38.1)

Perineural invasion (no. (%))	
Yes	41 (97.6)
No	1 (2.4)

TNM, *n* (%)	
I	0 (0)
II	26 (61.9)
III	7 (16.7)
IV	9 (21.4)

Elevated serum CA19-9	
Yes	38 (90.5)
No	4 (9.5)

Elevated serum CA125	
Yes	14 (33.3)
No	28 (66.7)

**Table 2:** CD86 positive cells infiltration in primary tumors compared to metastases.

	Samples	CD86		*Z*	*P* value^∗^
		Range	Median		
Primary tumor	42	2–31.2	11.6	−2.513	**0.012**
Metastasis	50	1.4–27.8	8.4		

Bold values indicate statistically significant correlations with *P* values less than 0.05. ^∗^Mann–Whitney *U* test.

**Table 3:** CD163 positive cells infiltration in primary tumors compared to metastases.

	Samples	CD163		*Z*	*P* value^∗^
		Range	Median		
Primary tumor	42	7.6–38.2	27.1	−2.175	**0.030**
Metastasis	50	8.4–45	31.0		

Bold values indicate statistically significant correlations with *P* values less than 0.05. ^∗^Mann–Whitney *U* test.

**Table 4:** Relationship between CD86, CD163, and clinicopathological parameters in 42 primary tumors of PDAC.

Clinicopathological parameters	CD86		*P* value^∗^	CD163		*P* value^∗^
	High	Low		High	Low	
Gender			0.217			0.064
Male	9	13		8	14	
Female	12	8		13	7	

Maximum diameter of tumor (cm)			0.739			0.739
≤4	15	15		6	7	
>4	7	6		15	14	

Grade			0.123			0.355
High/moderate differentiation	8	13		9	12	
Poor differentiation	13	8		12	9	

Vein invasion			1.000			0.525
Yes	13	13		12	14	
No	8	8		9	7	

Perineural invasion			1.000			1.000
Yes	21	20		21	20	
No	0	1		0	1	

TNM stage			0.204			0.204
I-II	11	15		15	11	
III-IV	10	6		6	10	

Elevated serum CA199			0.599			1.000
Yes	18	20		19	19	
No	3	1		2	2	

Elevated serum CA125			0.513			0.513
Yes	6	8		8	6	
No	15	13		13	15	

^∗^Chi-square test and Fisher's exact test.

**Table 5:** PD-L1 expression in primary tumors compared to metastases.

	Samples	PD-L1		*Z*	*P* value^∗^
		Range	Median		
Primary tumor	42	0–50%	0	−0.726	0.468
Metastasis	50	0–20%	0		

^∗^Mann–Whitney *U* test.

**Table 6:** Relationship between PD-L1 and clinicopathological parameters in 42 primary tumors of PDAC.

Clinicopathological parameters	PD-L1		*P* value^∗^
	Positive	Negative	
Gender			0.845
Male	6	16	
Female	6	14	

Maximum diameter of tumor (cm)			0.874
≤4	9	20	
>4	3	10	

Grade			0.063
High/moderate differentiation	3	17	
Poor differentiation	9	13	

Vein invasion			1.000
Yes	7	19	
No	5	11	

Perineural invasion			1.000
Yes	12	29	
No	0	1	

TNM stage			**0.031**
I-II	11	15	
III-IV	1	15	

Elevated serum CA199			1.000
Yes	11	27	
No	1	3	

Elevated serum CA125			1.000
Yes	4	10	
No	8	20	

Bold values indicate statistically significant correlations with *P* values less than 0.05. ^∗^Chi-square test and Fisher's exact test.

**Table 7:** Relationship between E-cadherin, N-cadherin. and clinicopathological parameters in 42 primary tumors of PDAC.

Clinicopathological parameters	E-cadherin		*P* ^∗^	N-cadherin		*P* ^∗^	Snail		*P* ^∗^
	High expression	Low expression		Positive	Negative		High expression	Low expression	
Gender			0.890			0.231			**0.002**
Male	19	3		5	17		5	14	
Female	16	4		1	19		17	6	

Diameter (cm)			0.765			1.000			0.936
≤4	25	4		4	25		13	16	
>4	10	3		2	11		6	1	

Grade			1.000			**0.037**			0.226
High/Moderate	17	3		0	20		11	9	
Poor	18	4		6	16		8	14	

Vein invasion			0.320			0.476			0.987
Yes	20	6		5	21		7	9	
No	15	1		1	15		11	14	

Perineural invasion			0.167			1.000			0.452
Yes	35	6		6	35		18	23	
No	0	1		0	1		1	0	

TNM stage			0.118			1.000			**0.047**
I-II	24	2		4	22		9	17	
III-IV	11	5		2	14		10	5	

Elevated serum CA199			0.532			0.474			1.000
Yes	32	6		5	33		17	21	
No	3	1		1	3		2	2	

Elevated serum CA125			0.306			**0.019**			0.273
Yes	10	4		5	9		8	6	
No	25	3		1	27		11	17	

Bold values indicate statistically significant correlations with*P* values less than 0.05. ^∗^Chi-square test and Fisher's exact test.

## Data Availability

The data can be supplied with appropriate request from the corresponding author.
